# Coverage of Nonpharmacologic Treatments for Low Back Pain Among US Public and Private Insurers

**DOI:** 10.1001/jamanetworkopen.2018.3044

**Published:** 2018-10-05

**Authors:** James Heyward, Christopher M. Jones, Wilson M. Compton, Dora H. Lin, Jan L. Losby, Irene B. Murimi, Grant T. Baldwin, Jeromie M. Ballreich, David A. Thomas, Mark C. Bicket, Linda Porter, Jonothan C. Tierce, G. Caleb Alexander

**Affiliations:** 1 Center for Drug Safety and Effectiveness, Johns Hopkins Bloomberg School of Public Health, Baltimore, Maryland; 2 Department of Epidemiology, Johns Hopkins Bloomberg School of Public Health, Baltimore, Maryland; 3 Office of the Assistant Secretary for Planning and Evaluation, US Department of Health and Human Services, Washington, DC; 4 National Institute on Drug Abuse, National Institutes of Health, Bethesda, Maryland; 5 Centers for Disease Control and Prevention, Division of Unintentional Injury Prevention, Atlanta, Georgia; 6 Department of Health Policy & Management, Johns Hopkins Bloomberg School of Public Health, Baltimore, Maryland; 7 Department of Anesthesiology and Critical Care Medicine, Johns Hopkins School of Medicine, Baltimore, Maryland; 8 National Institute of Neurological Disorders and Stroke, National Institutes of Health, Bethesda, Maryland; 9 Division of General Internal Medicine, Johns Hopkins Medicine, Baltimore, Maryland

## Abstract

**Importance:**

Despite epidemic rates of addiction and death from prescription opioids in the United States, suggesting the importance of providing alternatives to opioids in the treatment of pain, little is known regarding how payers’ coverage policies may facilitate or impede access to such treatments.

**Objective:**

To examine coverage policies for 5 nonpharmacologic approaches commonly used to treat acute or chronic low back pain among commercial and Medicare Advantage insurance plans, plus an additional 6 treatments among Medicaid plans.

**Design, Setting, and Participants:**

Cross-sectional study of 15 commercial, 15 Medicaid, and 15 Medicare Advantage health plans for the 2017 calendar year in 16 states representing more than half of the US population. Interviews were conducted with 43 senior medical and pharmacy health plan executives from representative plans.

**Main Outcomes and Measures:**

Medical necessity and coverage status for the treatments examined, as well as the use of utilization management tools and cost-sharing magnitude and structure.

**Results:**

Commercial and Medicare insurers consistently regarded physical and occupational therapy as medically necessary, but policies varied for other therapies examined. Payers most commonly covered physical therapy (98% [44 of 45 plans]), occupational therapy (96% [43 of 45 plans]), and chiropractic care (89% [40 of 45 plans]), while transcutaneous electrical nerve stimulation (67% [10 of 15 plans]) and steroid injections (60% [9 of 15 plans]) were the most commonly covered among the therapies examined for Medicaid plans only. Despite evidence in the literature to support use of acupuncture and psychological interventions, these therapies were either not covered by plans examined (67% of all plans [30 of 45] did not cover acupuncture) or lacked information about coverage (80% of Medicaid plans [12 of 15] lacked information about coverage of psychological interventions). Utilization management tools, such as prior authorization, were common, but criteria varied greatly with respect to which conditions and what quantity and duration of services were covered. Interviewees represented 6 Medicaid managed care organizations, 2 Medicare Advantage or Part D plans, 9 commercial plans, and 3 trade organizations (eg, Blue Cross Blue Shield Association). Interviews with plan executives indicated a low level of integration between the coverage decision-making processes for pharmacologic and nonpharmacologic therapies for chronic pain.

**Conclusions and Relevance:**

Wide variation in coverage of nonpharmacologic treatments for low back pain may be driven by the absence of best practices, the administrative complexities of developing and revising coverage policies, and payers’ economic incentives. Such variation suggests an important opportunity to improve the accessibility of services, reduce opioid use, and ultimately improve the quality of care for individuals with chronic, noncancer pain while alleviating the burden of opioid addiction and overdose.

## Key Points

**Question:**

Among US insurers, what are the coverage and utilization management policies for nonpharmacologic treatments for chronic, noncancer low back pain?

**Findings:**

In this cross-sectional study of 45 Medicaid, commercial, and Medicare Advantage plans, most plans covered at least physical and occupational therapy and chiropractic care for chronic noncancer pain, but there was little evidence of coverage of acupuncture and psychological interventions. Utilization management strategies such as visit limits and prior authorization were common, but criteria varied widely across the plans examined.

**Meaning:**

The lack of consistent coverage and utilization management policies underscores the need for best practices to improve comprehensive, multimodal coverage of treatments for chronic, noncancer low back pain.

## Teaser

This cross-sectional study examines rates of coverage and utilization management policies for nonpharmacologic treatments for chronic, noncancer low back pain among 45 Medicaid, Medicare Advantage, and commercial 2017 health insurance plans from 16 US states.

## Introduction

Opioid overdose deaths in the United States have risen to epidemic proportions, driven by an approximate 4-fold increase in prescription opioid sales that occurred between 1999 and 2010.^1,2^ While deaths from heroin and highly potent synthetic opioids such as illicit fentanyl have increased dramatically since 2010, prescription opioids remain a major contributor to overdose deaths in the United States. In 2016, 42 249 people died from overdoses due to prescription or illicit opioids in the United States, with 17 087 deaths attributed to prescription opioids^3,4^ and more than 2 million individuals estimated to have a prescription opioid use disorder.^5^ Projections suggest that an even greater number of Americans will be found to have died from an opioid overdose in 2017.^6^

Although many factors have contributed to increased use of opioids during the past 2 decades, 1 important contributor has been their overuse for the treatment of chronic, noncancer pain in the absence of data demonstrating long-term benefit.^7,8,9^ This is important because opioids are but 1 of many pharmacologic and nonpharmacologic treatments for pain from chronic conditions such as lower back pain, headache, and fibromyalgia. In 2016, the Centers for Disease Control and Prevention released its Guideline for Prescribing Opioids for Chronic Pain,^10^ recommending the use of nonopioid and nonpharmacologic therapies as first-line treatment for chronic pain. Consistent with other recent clinical practice guidelines,^11,12,13^ the Centers for Disease Control and Prevention also advises that if opioids are prescribed, they should be combined with nonpharmacologic and nonopioid therapies. An increasing volume of evidence and consensus demonstrates the role of many of these approaches in clinical practice, underscoring the opportunities that exist to simultaneously improve the quality of care for those with pain while reducing exposure to and overreliance on prescription opioids.^14,15,16,17^

Recognizing the importance of nonopioid therapies, the US Department of Health and Human Services’ National Pain Strategy urges changes to insurers’ coverage policies to enable greater access and adherence to nonopioid therapies for pain.^9^ Although coverage policies are an important determinant of health services utilization, little is known regarding payers’ coverage of nonpharmacologic treatments for chronic, noncancer pain. This information gap constrains the policy development and implementation process.

To address this gap, we examined 2017 policies for these treatment modalities for low back pain—1 of the most common pain-related conditions—among a diverse sample of 45 commercial, Medicaid, and Medicare Advantage plans. We focused on 5 common nonpharmacologic therapies for pain across all plans (physical therapy, occupational therapy, chiropractic care, acupuncture, and therapeutic massage), and, owing to the greater availability of information in public Medicaid coverage documents, examined an additional 6 therapies among Medicaid plans (transcutaneous electrical nerve stimulation [TENS], psychological interventions, steroid injections, facet injections, laminectomy, and diskectomy). We also conducted key informant interviews with 43 senior executives from the examined plans to better understand how plans have determined coverage and utilization management criteria for the treatments of interest. The aim of the study was to describe the current landscape of coverage for these therapies, and we did not conduct any formal hypothesis testing or infer causal pathways regarding coverage policies among the sampled plans.

## Methods

### Plan Selection

Our approach has been previously described.^18^ Briefly, we selected 45 plans from 16 different states using a strategy that was designed to achieve broad geographic diversity across the United States while also including states that have been differentially affected by the opioid epidemic and that vary with respect to state wealth. Whereas our first published analysis, of pharmacologic coverage,^17^ focused on 50 plans, including 20 commercial plans, 5 of those 20 commercial plans did not have available information about nonpharmacologic treatments or were medication-only plans; these were excluded from this analysis. Our study follows the Strengthening the Reporting of Observational Studies in Epidemiology (STROBE) reporting guideline and Standards for Reporting Qualitative Research (SRQR) reporting guideline.

We placed all 50 states into tertiles of low, medium, and high Federal Medical Assistance Percentages scores, which are a proxy for the wealth of a state. We then selected 4 to 6 states with different geographic regions and population sizes within each tertile. We selected commercial and Medicare Advantage plans of different sizes across the country using enrollment data from the Kaiser Family Foundation and Medicare.gov. From each of 7 states, we selected 2 or 3 commercial plans for both large and small group employers. The minimum threshold for plan inclusion was information about pharmacologic coverage, as this was the focus of the first phase of the project. We included states (Figure) that in aggregate account for more than half of the US population. The eAppendix in the Supplement details our full plan selection process and rationale and eTable 1 in the Supplement contains the final list of selected plans.

**Figure.**
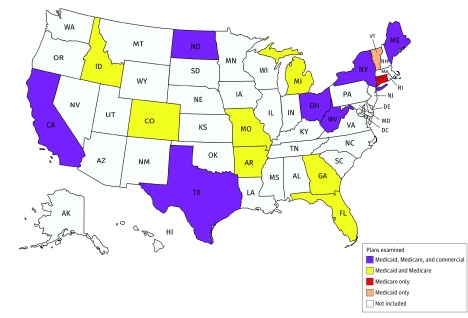
Geographic Diversity of Included Plans The figure was created with mapchart.net.

### Selection of Nonpharmacologic Treatments

We used a sequential process to select our treatments of interest. First, we derived a list of potential treatments based on our own knowledge of nonpharmacologic treatments most commonly used for back pain. Next, we consulted with pain management researchers to refine our list based on standard practice and recommendations from clinical guidelines before triangulating the selection of therapies in consultation with an expert pain clinician (eTable 2 in the Supplement).

### Data Extraction

Our protocol was based on an earlier pilot study performed from May 5 through September 14, 2016, during which we examined coverage policy documents from 1 of the largest commercial insurance plans (Anthem) and Medicaid programs (Medi-Cal) in the United States. The pilot study allowed for us to refine our treatment list, gain familiarity with the publicly available variables in plan documents, and design data extraction forms and quality controls such as dual extraction and comparison of a subsample of data to ensure accuracy.

For this larger investigation, we searched for health plan–specific coverage documents that were publicly available on the internet for each plan of interest. These documents included the 2017 medical policy, summary of benefits and coverage, and evidence of coverage documents, or analogous documents with plan benefits.

Although criteria may vary from plan to plan, health care services or supplies considered medically necessary are generally defined as those needed to diagnose or treat a medical condition and meet accepted standards of medicine.^19^ Medical necessity determinations for treatments are made based on evidence of safety and efficacy as well as their relative efficiency and cost-effectiveness. Coverage policies and determinations of medical necessity are a mixture of national or regional policy guidelines that may or may not be modified at the state or local level depending on the payer.^20^ Thus, we first examined the national medical policies of 8 commercial payers, representing the commercial plans of interest in our study, and the Centers for Medicare & Medicaid Services for Medicare Advantage plans regarding the medical necessity of the 11 treatments of interest. Medical policies for Medicaid programs are made at the state level, so no equivalent national-level coverage determination exists for these plans; therefore, we did not include Medicaid plans in this phase of the analysis. We did not review the published evidence for or against these individual treatments, only documenting whether they were considered medically necessary.

We then assessed the coverage and management of these treatments among individual plans, as local coverage and utilization management determinations might diverge from national medical necessity policies. The information we extracted included health plan characteristics; nonpharmacologic coverage policies, including the use of utilization management strategies such as prior authorization, step therapy, and visit limits; and cost-sharing structures. To examine cost sharing, we abstracted the co-payments and co-insurances by treatment and preferred vs nonpreferred provider network for each plan.

Each policy document was reviewed and abstracted by a single reviewer, with accuracy validated through double-extraction and comparison by a second extractor for the first 20% of data.

### Key Informant Interviews

We supplemented our quantitative analyses with 20 key informant interviews with a total of 43 senior executives within many of the plans of interest. We focused on individuals responsible for the design, implementation, and evaluation of medical and pharmacy policy within the payer, such as chief medical officers, chief pharmacy officers, and vice presidents of clinical operations. Individuals were contacted and interviewed by telephone using a semistructured interview guide that was developed and iteratively piloted and pretested. Interviewees were assured confidentiality, and that their name, position title, and organization would not be disclosed beyond the study team without their explicit consent. An interviewer and research analyst were present for each call, taking extensive notes along with verbatim transcriptions of illustrative comments. Interview notes and verbatim quotes were shared and iterated between the interviewer and analyst directly following each interview in order to ensure completeness and accuracy. To the same end, occasionally the study team contacted interviewees in order to clarify or expand upon topics covered during the interview. The interview was based on 5 key domains: (1) plan responses to the opioid epidemic; (2) coordination between pharmacologic and nonpharmacologic pain therapies; and the development of (3) innovative strategies, (4) requirements, and (5) technologies to improve the care of patients with chronic, noncancer pain.

Our study was exempted from review by the Johns Hopkins Bloomberg School of Public Health institutional review board, and our interviewees participated with assurance of no attribution of their comments to them or their health plan.

### Data Analysis

We reviewed the data for entry errors or spurious values before visually inspecting the data distributions. Our primary outcomes of interest were medical necessity, coverage status, presence and specifications of utilization management tools, and cost-sharing magnitude and structure. We defined a treatment as covered if coverage documents explicitly stated that it was covered by the plan and as not covered if documents explicitly stated that it was not covered; otherwise, we classified it as unclear/not found. We used descriptive statistics to characterize outcomes across treatments and payer types.

To analyze our key informant interviews, we used a grounded theory approach.^21^ Interviews were analyzed in a sequential fashion. First, we reviewed and consolidated the notes from the interviewer and research analyst into a single document for each interview. Second, we organized each interviewees’ comments around our 5 study domains. Third, we generated a new study document that, for a given query, aggregated the diversity of feedback that we received regarding the topic in the interviews. Fourth, we synthesized this feedback in narrative form. This was an iterative process that was performed with several members of the investigational team, and early interview guides were modified in order to maximize the value of the qualitative information received, although the 5 core domains of interest remained consistent. In addition, illustrative quotes were identified and extracted throughout this process to support the insights derived.

## Results

### Medical Necessity

#### Commercial Plans

Physical and occupational therapy were frequently and consistently regarded as medically necessary treatments for low back pain by commercial insurers (Table 1). By contrast, of commercial payers where national documentation was available, medical necessity was inconsistently determined for treatments such as acupuncture (3 payers regarding as necessary; 0, necessary with preconditions; and 2, not necessary), TENS (3, necessary; 1, necessary with preconditions; and 2, not necessary), chiropractic care (2, necessary; 2, necessary with preconditions; and 0, not necessary), and facet injections (3, necessary; 0, necessary with preconditions; and 3, not necessary). Massage therapy was determined as not medically necessary by all payers. Coverage policy documents regarding the medical necessity of psychological interventions were not identified for any of the 8 commercial payers.

**Table 1.** Policies on Medical Necessity of Therapies for Medicare Advantage and 8 Commercial Plans

Pain Therapy	Medicare Advantage	Commercial Insurers, No. of Plans (n = 8)			
		Yes	Conditional^a^	No	Not Found
Rehabilitative therapies					
Physical therapy	Yes	8	0	0	0
Occupational therapy	Yes	7	0	0	1
Chiropractic care	Conditional	2	2	0	4
Acupuncture	No	3	0	2	3
Psychological interventions	Not found	0	0	0	8
Therapeutic massage	Not found	0	0	4	4
Transcutaneous electrical nerve stimulation	Yes	3	1	2	2
Injections					
Steroid	Yes	3	2	1	2
Facet	Yes	3	0	3	2
Back surgery					
Laminectomy	Conditional	0	3	1	4
Diskectomy	No	0	3	1	4

^a^ Conditional therapy is considered medically necessary depending on the condition causing back pain or the type of pain (eg, nonradicular pain).

#### Medicare Advantage Plans

Medical policies of the Centers for Medicare & Medicaid Services governing Medicare Advantage plans were similar to those of the commercial plans reviewed for physical therapy, occupational therapy, TENS, and steroid injections. They differed from commercial plans on facet injections, which they considered to be medically necessary, and acupuncture and diskectomy, which they did not consider medically necessary, contrary to the policies of many of the commercial plans. The medical necessity of chiropractic care was conditional upon the specific condition and health care professional type, which accorded with 2 of the 4 commercial insurers for which policies were available, and contrasted with the other 2, which considered it medically necessary. Information about the medical necessity of psychological interventions for pain was not available.

### Coverage and Utilization Management 

#### All Payers

Table 2 and Table 3 depict the coverage and utilization management of the examined therapies across the 45 Medicaid, commercial, and Medicare Advantage plans examined. Across all 3 payers, physical therapy was the most commonly covered (98% [44 of 45 plans]), followed by occupational therapy (96% [43 of 45 plans]) and chiropractic care (89% [40 of 45 plans]). We rarely found evidence of coverage for acupuncture (20% [9 of 45 plans]), and most plans explicitly did not cover it (67% [30 of 45 plans]). Therapeutic massage was almost never covered (2% [1 of 45 plans]), although in this case most plans had no information available about coverage status (78% [35 of 45 plans]). Utilization management tools were common for the therapies examined, and visit limits were the most common, especially for rehabilitative therapies, including physical therapy (55% [24 of 44 plans]), occupational therapy (53% [23 of 43 plans]), and chiropractic care (50% [20 of 40 plans]). Likewise, prior authorization was also relatively common for covered therapies, including for physical therapy (23% [10 of 44 plans]), occupational therapy (21% [9 of 43 plans]), and chiropractic care (8% [3 of 40 plans]). The relative frequency of utilization management strategies varied considerably by therapy and by payer type.

**Table 2.** Coverage and Utilization Management for Nonpharmacologic Pain Therapies in 15 Medicaid Plans

Pain Therapy	No. of Plans						
	Coverage			Utilization Management			
	Covered	Unclear or Not Found	Not Covered	Prior Authorization	Condition Requirements	Visit Limits	Referral Requirements
Rehabilitative therapies							
Physical therapy	14	1	0	4	2	14	10
Occupational therapy	14	1	0	3	3	14	10
Chiropractic care	12	2	1	1	9	11	1
Acupuncture	2	5	8	1	1	1	0
Therapeutic massage	1	10	4	1	1	1	0
Psychological interventions	3	12	0	0	0	0	0
Transcutaneous electrical nerve stimulation	10	5	0	7	1	2	3
Injections							
Steroid	9	6	0	3	1	3	0
Facet	7	8	0	2	1	1	0
Back surgery							
Laminectomy	3	12	0	1	0	0	0
Diskectomy	2	13	0	1	0	0	0

**Table 3.** Coverage and Utilization Management for Select Nonpharmacologic Pain Therapies in 15 Commercial and 15 Medicare Advantage Plans

Pain Therapy	No. of Plans						
	Coverage			Utilization Management			
	Covered	Unclear or Not Found	Not Covered	Prior Authorization	Condition Requirements	Visit Limits	Referral Requirements
**Commercial**							
Rehabilitative therapies							
Physical therapy	15	0	0	1	0	10	1
Occupational therapy	14	1	0	1	0	9	1
Chiropractic care	13	1	1	1	0	8	0
Acupuncture	3	1	11	0	0	1	0
Therapeutic massage	0	12	3	0	0	0	0
**Medicare Advantage**							
Rehabilitative therapies							
Physical therapy	15	0	0	5	0	0	1
Occupational therapy	15	0	0	5	0	0	1
Chiropractic care	15	0	0	1	12	1	0
Acupuncture	4	0	11	0	1	2	0
Therapeutic massage	0	13	2	0	0	0	0

#### Medicaid Plans

Table 2 depicts the coverage and utilization management of 11 nonpharmacologic treatments among the 15 Medicaid plans we examined. Among the therapies examined across Medicaid, Medicare Advantage, and commercial plans, physical therapy (93% [14 of 15 plans]), occupational therapy (93% [14 of 15 plans]), and chiropractic care (80% [12 of 15 plans]) were the most frequently covered. Acupuncture (13% [2 of 15 plans]) and therapeutic massage (7% [1 of 15 plans]) were rarely covered. Among the 6 therapies examined among Medicaid plans only, TENS (67% [10 of 15 plans]) was most commonly covered, followed by steroid injections (60% [9 of 15 plans]), facet injections (47% [7 of 15 plans]), psychological interventions (20% [3 of 15 plans]), laminectomy (20% [3 of 15 plans]), and diskectomy (13% [2 of 15 plans]), although for the latter 3, information about coverage status was predominantly unavailable (80% [12 of 15 plans] for psychological interventions and laminectomy and 87% [13 of 15 plans] for diskectomy).

Utilization management tools were common for the therapies examined in Medicaid plans. Visit limits were the most common utilization management tool, and rates were highest for the rehabilitative therapies, including physical therapy (100% [14 of 14 plans]), occupational therapy (100% [14 of 14 plans]), and chiropractic care (92% [11 of 12 plans]), and were less common for steroid injections (33% [3 of 9 plans]) and TENS (20% [2 of 10 plans]). Prior authorization requirements were also common, and were highest for TENS (70% [7 of 10 plans]), acupuncture (50% [1 of 2 plans]), steroid injections (33% [3 of 9 plans]), facet injections (29% [2 of 7 plans]), physical therapy (29% [4 of 14 plans]), and occupational therapy (21% [3 of 14 plans]).

Some therapies were subject to narrow condition requirements restricting services to patients with a specific diagnosis, or referral requirements necessitating that a specific provider (eg, primary care physician) write a prescription for a particular treatment. Condition requirements were most common for chiropractic care (75% [9 of 12 plans]), which was usually restricted to patients with spinal subluxation, and were used also for occupational therapy (21% [3 of 14 plans]) and physical therapy (14% [2 of 14 plans]). Referral requirements were also used for physical therapy (71% [10 of 14 plans]), occupational therapy (71% [10 of 14 plans]), and TENS (30% [3 of 10 plans]).

#### Commercial and Medicare Advantage Plans

Table 3 depicts the coverage and utilization management of the 5 nonpharmacologic treatments in the 15 commercial and 15 Medicare Advantage plans examined. Across both payer types, physical therapy (30 plans), occupational therapy (29 plans), and chiropractic care (28 plans) were the most commonly covered, followed by acupuncture (7 plans total, including 3 commercial and 4 Medicare Advantage plans). Therapeutic massage was not covered by any plans, but we were unable to locate coverage information regarding this therapy for 25 plans.

Utilization management tools were less prevalent, generally, among these plans compared with the Medicaid plans examined. Visit limits were common in the commercial plans, as in Medicaid plans, and rates were highest for physical therapy (67% [10 of 15 plans]), occupational therapy (64% [9 of 14 plans]), and chiropractic care (62% [8 of 13 plans]), followed by acupuncture (33% [1 of 3 plans]). Prior authorization requirements were rare among the commercial plans, and among Medicare Advantage plans they were only common for physical therapy (33% [5 of 15 plans]) and occupational therapy (33% [5 of 15 plans]), similar to the rates in Medicaid plans. Both condition and referral requirements were almost never evident among commercial and Medicare Advantage plans, except for chiropractic care in Medicare Advantage plans (75% [12 of 15 plans]), which was restricted to patients with spinal subluxation.

### Key Informant Interviews

We interviewed 43 senior executives from 20 organizations, including 6 Medicaid managed care organizations, 9 commercial plans, 2 Medicare Advantage or Part D plans, and 3 insurance trade associations (eg, Blue Cross/Blue Shield Association). The informants represented experts in medical policy, including 9 chief medical officers or medical directors, directors of behavioral health policy and plan design, and 10 directors of pharmacy policy. The key informant interviews included a focus on plans’ coverage policies for nonpharmacologic pain treatments in response to the opioid epidemic. Informants familiar with coverage decisions confirmed that they have undertaken strategies to expand access to nonpharmacologic therapies in recent years. For example, a trade association representative stated that his organization was observing more emphasis among plans on providing access to nonpharmacologic treatments such as physical therapy, chiropractic care, mindfulness training, and other nonopioid alternatives. However, with the exception of 1 Medicaid plan that initiated acupuncture coverage due to patient demand, informants also emphasized that new or emerging treatments were assessed using a formal, enterprise-wide, evidence-based protocol. Evaluation of nonpharmaceutical treatments was commonly based on standardized protocols such as those developed through the Medicaid Evidence-Based Decisions project or the Blue Cross Blue Shield Technology Evaluation and Coverage Program. Nearly all of the plans that we spoke with reported the importance of medical necessity when evaluating nonpharmacologic treatments for pain. Some plans incorporated additional elements of review through engagement with physician networks, community members, and other stakeholders.

Overall, informants indicated a low level of integration between coverage decision making for nonpharmacologic and pharmacologic therapies, such as through the use of step therapy requirements that encourage use of physical therapy before initiation of long-acting or extended-release opioids. There were some plans, however, that attempted to increase coordination through close cooperation between the individuals formulating pharmacy and medical policies. For example, 1 large commercial plan mentioned that the clinical policy director was also a member of the plan’s pharmacy and therapeutics committee. A large Medicaid program in a state disproportionately affected by the epidemic had fully integrated nonpharmacologic therapies into their step therapy requirements for opioid initiation. However, the majority of plans reported difficulty coordinating step therapy requirements between pharmacologic and nonpharmacologic therapies.

Interviewees provided several important insights regarding novel requirements, technologies, and innovative strategies to combat the opioid epidemic. However, this information centered predominantly on improved formulary management for opioids, detection and engagement with overprescribers and doctor shoppers, and improved access to treatment for substance use disorders, and less so on innovations aimed at optimizing coverage and access to nonpharmacologic therapies for chronic pain. Changes to coverage policies for analgesic drugs, including opioids, were the most common element of plans’ responses to the opioid epidemic.

Several barriers to expanding coverage of nonpharmacologic treatment options for chronic, noncancer pain were mentioned by interviewees, including an inadequate evidence base, cost concerns, and the potential for inappropriate use of some therapies, such as yoga and therapeutic massage. Some informants mentioned the increased administrative burden of expanded coverage, such as the resources required to process prior authorization and step therapy requirements for new therapies. For example, 1 interviewee mentioned wanting step therapy for steroid injections, but that the extra paperwork would require the services of an outside vendor. Interviewees also noted the potential for adverse selection should competitors choose not to expand coverage in a similar manner.

### Variation in Utilization Management Criteria Across Plans

There was wide variation in utilization management criteria for specific treatments across the plans we examined (eTable 3 in the Supplement). For example, plans with utilization management for physical therapy varied with respect to qualifying conditions, prescription requirements, the number of visits covered, the duration limit of a single prescription, the type of provider network, and expectations regarding improvement in functioning and recovery. Similar variation was observed with respect to utilization management criteria for chiropractic care and acupuncture. Notably, the qualifying conditions for these therapies varied among plans, with chiropractic care approved for spinal subluxation only in the majority of plans, but sometimes approved for “musculoskeletal disorders” or “mechanical/myofascial extremity pain or structural imbalance, distortion or subluxation in the human body,” while acupuncture was approved variously for chronic pain lasting in duration for less than 3 months, more than 3 months, or for nausea only.

For physical therapy, visit limits imposed by plans ranged from 15 to 30 visits per year, with some plans allowing between 40 and 60 visits per year combined with occupational and/or speech therapy. Duration limits for each prescription varied as well, ranging from 60 days to 1 year. The same trend was apparent for chiropractic care, which was restricted, variously, to between 6 and 30 visits per year by specific plans. Plans variously restricted some therapies to minutes per day, or to units or dollar amounts per year, particularly among Medicaid plans.

### Out-of-Pocket Costs

Table 4 illustrates the median in-network co-payment and the median out-of-network co-insurance per visit for each therapy covered by Medicare Advantage or commercial plans among plans for which information was available. Cost-sharing structures differed by insurer and by plan type, with some plans structuring costs according to whether the provider was in or out of network, or whether there was a deductible, and, if so, whether it had been met. Median in-network co-payments were generally similar between Medicare Advantage and commercial plans for physical therapy ($40 vs $30) and occupational therapy ($40 vs $38). Commercial plans required much higher co-payments and co-insurance rates than Medicare Advantage plans for chiropractic care ($60 vs $20 in network; 50% vs 35% out of network) and acupuncture ($43 vs $8 in network; 50% vs no out-of-network Medicare Advantage coverage).

**Table 4.** Median Out-of-Pocket Costs of Covered Nonpharmacologic Therapies

Pain Therapy	Median (IQR)			
	Medicare Advantage		Commercial	
	In Network (Co-payment), $	Out of Network (Co-insurance) %	In Network (Co-payment), $	Out of Network (Co-insurance) %
Physical therapy	40.0 (32.5-40.0)	30^a^	30.0 (15.0-50.0)	30 (23-38)
Occupational therapy	40.0 (32.5-40.0)	30^a^	38.0 (18.8-52.5)	25 (20-30)^b^
Chiropractic care	20.0 (17.5-20.0)	35 (33-38)^c^	60.0^a^	50^a^
Acupuncture	8.0 (0-20)^b^	NA	43.0 (26.3-58.8)^c^	50^a^

Abbreviations: IQR, interquartile range: NA, not applicable.

^a^ Based on information from 1 plan.

^b^ Based on information from 4 plans.

^c^ Based on information from 2 plans.

## Discussion

To our knowledge, this is the first large-scale assessment of a diverse group of insurers’ coverage policies regarding nonpharmacologic treatments for chronic, noncancer pain in the opioid era. We examined the coverage and management of several commonly used treatment modalities for low back pain among a representative sample of 45 commercial, Medicaid, and Medicare Advantage health plans. While there was near universal coverage of physical and occupational therapy across insurers, coverage of other nonpharmacological treatments such as acupuncture and psychological interventions was less consistent. Further, prior authorization and other restrictions such as visit limits were quite common, and there was noteworthy variation in the utilization management criteria imposed across different plans and payer types. These findings are important given the growing literature indicating that nonpharmacologic treatments for chronic, noncancer pain can reduce opioid exposure and overuse and improve patients’ quality of care and pain control.^10,11,12,13,14,15,16,17^

Despite a growing evidence base supporting the effectiveness and cost-effectiveness of many of the nonpharmacological treatments examined in our study, our findings depict inconsistent and often absent coverage for many of these treatments. Given recommendations in recent clinical practice guidelines that prioritize nonopioid treatments, including nonpharmacological treatments, as first-line therapy for many types of chronic pain, including low back pain, our findings highlight a number of opportunities across a broad range of public and private payers to improve coverage and reimbursement policies for nonpharmacological treatments for pain. For example, the out-of-pocket expense for nonpharmacologic treatments may be prohibitive for some patients; in contrast with pharmacologic treatments, which typically require just 1 co-payment per prescription, treatment-based approaches can require a co-payment for each visit, in addition to costs associated with travel and missed work. These issues are multiplied if a patient is taking a multipronged approach that incorporates multiple therapies for chronic pain. In addition, the wide variation in utilization management criteria that we identified underscores the uncertainty that may exist around what constitutes an appropriate duration and intensity of treatment (eg, physical therapy) for chronic, noncancer pain.

We did not find coverage information regarding many of the nonpharmacological treatments we examined. For example, among individual plans, coverage information was often unavailable for therapeutic massage and acupuncture, while among therapies examined for Medicaid plans only, information could not be found for diskectomy, laminectomy, psychological interventions, and facet injections. Furthermore, national medical policies also lacked information about the medical necessity of certain therapies, including psychological interventions, therapeutic massage, laminectomy, diskectomy, and acupuncture. This lack of information may itself serve as a barrier to treatment for both patients and clinicians. Interviews with key informants revealed a relative emphasis among plans on reducing opioid overuse as opposed to addressing pain management comprehensively and using multiple modalities, which may explain in part the lack of publicly available information about nonpharmacologic treatments.

Among the plans examined, coverage for certain treatments appeared to be inconsistent with the available evidence. For example, steroid injections were covered by nearly two-thirds of the Medicaid plans examined, and facet injections by nearly half, despite a lack of high-quality data supporting use of these interventions for low back pain.^17,22,23,24^ These therapies were also considered medically necessary for chronic low back pain by the Centers for Medicare & Medicaid Services, and for nearly half of the commercial plans examined. Conversely, although there are growing data to support the use of acupuncture for back pain, approximately half of public plans and three-fourths of private plans did not cover this intervention.^17,24^ Of particular interest was the finding that medical necessity for psychological interventions was not included in any payer documents examined despite a strong evidence base in favor of its use.^17,24^

Interviews with key informants provided several important insights about plans’ approaches to coverage and management of nonpharmacologic therapies for pain. Several interviewees mentioned an insufficient evidence base when it came to increased coverage of nonpharmacologic therapies, underscoring the need for additional research and evidence synthesis in order to make a case for coverage of these treatments. In addition, interviews revealed that the decision-making processes for coverage of pharmacologic and nonpharmacologic therapies remain largely siloed from one another, which suggests that plans may not be integrating coverage of nonpharmacologic therapies with pharmacologic treatment in the context of multimodal, comprehensive treatment for chronic, noncancer pain.

### Limitations

Our analysis is subject to several limitations. First, coverage policies alone do not ensure access to treatment, which is influenced by factors we did not measure, such as the size and coordination of plan networks and the availability and willingness of clinicians to provide these services in a given community. Second, our sample of 45 plans may not be representative of the national coverage landscape, despite efforts to create a representative sample. In addition, our analysis reflects payer policies at a single point in time, although payer plans continue to rapidly evolve, a fact made apparent by our key informant interviews. This speaks to the need for subsequent analyses of payer policies in order to measure the scope and impact of future policy changes. Furthermore, our conclusions are limited to the therapies selected for inclusion, which, although determined to be of the highest relevance by a multidisciplinary team of pain experts, do not represent all nonpharmacologic treatments for pain, including some likely to experience increased coverage in the future. For example, therapies such as interdisciplinary pain programs and group-based yoga and mindfulness are increasingly examined in clinical trials and have evidence of benefit. Finally, coverage documents frequently did not have details about prior authorization or visit limits, especially among commercial plans. Thus, we may underestimate the prevalence of such requirements. Cost information was also frequently unavailable; as a result, aggregate costs data represent a subset of the overall plans examined.

## Conclusions

Insurers are increasingly recognized as influential stakeholders that are well positioned to drive changes in pain treatment practices. One key component of such changes is the greater use of nonpharmacologic approaches to managing chronic, noncancer pain, as has been recommended by the Centers for Disease Control and Prevention,^10^ the President’s Commission on Combating Drug Addiction and the Opioid Crisis,^8^ and others.^25^ To our knowledge, our work represents the most comprehensive assessment of coverage policies regarding the medical necessity, coverage, and management of nonpharmacologic treatments for back pain. Although payers generally covered many nonpharmacologic therapies, medical necessity and coverage policies were sometimes inconsistent with relevant evidence syntheses.^10,14,15,16,17^ Furthermore, utilization management requirements were highly variable, which speaks to a need for evidence-based guidance regarding the optimal use of these therapies, and standardized, comprehensive training for practitioners to effectively implement the evidence base into their practice. While additional high-quality evidence of the efficacy of nonpharmacologic treatments is necessary, our evaluation can nevertheless be used to inform federal, state, and local efforts to reduce opioid-related injuries and deaths by improving patient access to nonpharmacologic pain therapies.

## Notes

Supplement. **eAppendix.** Selection of Plans **eTable 1.** Payers and Plans Examined **eTable 2.** List of Non-Pharmacologic Treatments **eTable 3.** Differential Incorporation of Utilization Management Strategies Across 45 Medicaid, Medicare Advantage and Commercial Plans for Select Covered Non-Pharmacologic Treatments **eReferences**
