# Supplementary material for: Coverage of Nonpharmacologic Treatments for Low Back Pain Among US Public and Private Insurers
Source: JAMA Netw Open. 2018 Oct 5;1(6):e183044. doi: 10.1001/jamanetworkopen.2018.3044 (PMC6324451; doi:10.1001/jamanetworkopen.2018.3044)
Supplement: Supplement. — eAppendix. Selection of Plans eTable 1. Payers and Plans Examined eTable 2. List of Non-Pharmacologic Treatments eTable 3. Differential Incorporation of Utilization Management Strategies Across 45 Medicaid, Medicare Advantage and Commercial Plans for Select Covered Non-Pharmacologic Treatments eReferences [file jamanetwopen-1-e183044-s001.pdf]

Supplementary Online Content

Heyward J, Jones CM, Compton WM, et al. Coverage of Nonpharmacologic Treatments for Low Back Pain Among US Public and Private Insurers. *JAMA Netw Open.* 2018;1(6):e183044. doi:10.1001/jamanetworkopen.2018.3044

eAppendix. Selection of Plans

eTable 1. Payers and Plans Examined

eTable 2. List of Non-Pharmacologic Treatments

eTable 3. Differential Incorporation of Utilization Management Strategies Across 45 Medicaid, Medicare Advantage and Commercial Plans for Select Covered Non-Pharmacologic Treatments

eReferences

This supplementary material has been provided by the authors to give readers additional information about their work.

© 2018 Heyward J et al. *JAMA Network Open.*

26

© 2018 Heyward J et al. *JAMA Network Open.*

eAPPENDIX. SELECTION OF PLANS.

Selection of Medicaid Plans

We selected 16 states based on varying demographics, such as large and small populations, wealth, level of urbanicity, and those with disproportionately high rates of injuries and deaths from prescription and non-prescription opioids, such as Ohio, West Virginia and Maine.[1] Within tertiles of FMAP scores, we selected 4-6 states with varying population sizes and geographic regions, as defined by the United States Census Bureau. As a result, we selected 1 to 2 states from each of the 9 geographic regions. Using data from the Kaiser Family Foundation,[2] we selected Medicaid formularies from the largest Managed Care Organization (MCO) in each state, with the exception of one state's largest MCO, whose publicly available formulary listed analgesics in a unique fashion. We instead used the formulary from the second largest Medicaid MCO in this state.

Selection of Medicare Advantage Plans

Since their inception, the proportion of Medicare beneficiaries enrolled in Medicare Advantage ("Part C") has steadily grown, relative to traditional ("fee-for-service") Medicare.[3] In 2017, Medicare Advantage enrollees numbered 19.0 million and accounted for 33% of all Medicare beneficiaries. To maximize representativeness, we selected the same 15 states as for the Medicaid plans, with the exception of Vermont, where we substituted Connecticut, due to Medicare enrollment data availability. Connecticut also has a relatively small population and is located in the same geographic region as Vermont. Our overall selection of states for Medicare Advantage also varied greatly in the number of their Medicare Advantage beneficiaries.

We referred to enrollment data from Medicare.gov to select Medicare Advantage Plan Types, such as health maintenance organization (HMO) or preferred provider organization (PPO) plans, with the largest or second largest enrollment in each state. Meanwhile, we ensured selection of plans from the largest 5 Medicare Advantage insurers, including Aetna, Anthem, Humana, Kaiser Foundation and United Healthcare, as well as a variety of smaller payers. We then selected plans with a variety of star ratings, which are offered for reference by medicare.gov. Star ratings are an evaluation of a plan's overall quality and performance, determined by the Centers for Medicare and Medicaid Services, while taking member experience into consideration.

Selection of Commercial Plans

We selected 15 plans derived from a total of 7 states, in order to examine multiple plans within the same state. These 7 states were of different population sizes and geographic regions, selected from the list of states for the Medicaid plans. These states also varied in the magnitude by which they were affected by the opioid epidemic. We examined three commercial plans from each of 2 states, two commercial plans from each of 4 states, and one commercial plan from the last state.

Recent estimates suggest 74.9 million Americans are covered under private insurance.[4] The majority (57%) of these are covered under what's considered large group plans (greater than 51 employees). The remaining individuals are covered under either small group plans (19.6%) or the individual markets (23.3%). We focus our analysis on the small and large group markets, given that these markets have been the predominant provider of private health insurance. To identify plans of interest, we used the Kaiser Family Foundation data on individual states and the largest enrollments in both the large and small group health insurance markets.[5] Of note, there is significant overlap of insurance carriers between the individual, small, and large group markets. For example, the top three carriers in California are the same across all three markets

with slight differences in ordering. Additionally, within the same insurance carrier, formularies do not vary much outside of cost-sharing levels, such that a UnitedHealthcare formulary in one state will be similar, if not identical, to the UnitedHealthcare formulary in another state. Once a potential insurance plan was identified, we examined the insurance carrier website for access to the specific state-level formulary. Some plans restricted the plan-specific documents and level of information made publicly available; in these cases, we selected a different plan with large enrollment, but in the same state.

© 2018 Heyward J et al. *JAMA Network Open.*

eTABLE 1. PAYERS AND PLANS EXAMINED.

State	Medicaid	Medicare Advantage	Commercial Plan
Arkansas	Arkansas Medicaid	United Healthcare	---
California	MediCal	Kaiser Foundation	Anthem Blue Cross Blue Shield
Colorado	Health First Colorado	Kaiser Foundation	---
Connecticut	---	Aetna	---
Florida	Florida Medicaid	Humana	---
Georgia	Georgia Medicaid	Humana	---
Idaho	Idaho Medicaid	Anthem	---
Maine	MaineCare	Martin's Point Generations Advantage	Aetna<br>Anthem
Michigan	Michigan Medicaid	Anthem	---
Missouri	MO HealthNet	Aetna	---
New York	New York Medicaid	Healthfirst	Aetna<br>Empire Blue Cross<br>Excellus BCBS
North Dakota	North Dakota Medicaid	Medica	Blue Cross Blue Shield North Dakota<br>Medica
Ohio	Ohio Medicaid	MediGold	Anthem<br>Medical Mutual of Ohio<br>United Healthcare
Texas	Texas Medicaid	United Healthcare	HCSC<br>United Healthcare
Vermont	Vermont Medicaid	---	---
West Virginia	West Virginia Medicaid	Aetna	Aetna<br>Highmark

© 2018 Heyward J et al. *JAMA Network Open.*

eTABLE 2. LIST OF NON-PHARMACOLOGIC TREATMENTS.

Medicaid	Medicare Advantage and Commercial
Acupuncture	Acupuncture
Chiropractic care	Chiropractic care
Occupational Therapy	Occupational Therapy
Physical Therapy	Physical Therapy
Therapeutic massage	Therapeutic massage
Transcutaneous electrical nerve stimulation (TENS)	
Psychological interventions	
Steroid injections	
Facet Injections	
Lumbar laminectomy	
Lumbar discectomy	

© 2018 Heyward J et al. *JAMA Network Open.*

eTABLE 3. DIFFERENTIAL INCORPORATION OF UTILIZATION MANAGEMENT STRATEGIES ACROSS 45 MEDICAID, MEDICARE ADVANTAGE AND COMMERCIAL PLANS FOR SELECT COVERED NON-PHARMACOLOGIC TREATMENTS

**Physical Therapy**	**Chiropractic Care**	**Acupuncture**
Condition requirements:<br>• Medically determinable functional physical impairment, weakness, atrophy, and/or a decreased range of motion<br>• Loss of function due to illness, injury, loss of a body part, or congenital abnormality<br><br>Quantity Limits:<br>• 75 min/day<br>• 2 hours/day<br>• 15 visits/year<br>• 20 visits/year<br>• 25 visits/year<br>• 30 visits/year<br>• 30 visits/year combined physical and speech therapy<br>• 40 visits/year combined physical and occupational therapy<br>• 60 visits/year combined physical, occupational, and speech therapy<br><br>Provider requirements:<br>• In-network provider<br>• Licensed provider<br>• Passed National Physical Therapy Examination (NPTE)<br><br>Duration Limit per prescription:<br>• 60 days<br>• 90 days<br>• 6 months<br>• 1 year<br><br>Prescription requirements:<br>• Written plan of care, including type of services, amount, frequency, duration and measurable goals<br>• Comprehensive evaluation to determine if PT is medically necessary<br><br>Other requirements:<br>• Services must require unique knowledge, skills and judgment of a physical therapist<br>• Services must be expected to result in improvement in functioning within reasonable time	Condition requirements:<br>• Musculoskeletal disorders<br>• Subluxation only<br>• Mechanical/myofascial extremity pain or structural imbalance, distortion or subluxation in the human body<br><br>Quantity Limits:<br>• 2 visits/month<br>• 6 visits/year<br>• 10 visits/year<br>• 12 visits/year<br>• 16 visits/year combined chiropractic care and acupuncture<br>• 20 visits/year<br>• 30 visits/year<br>• 35 visits/year<br><br>Provider requirements:<br>• Licensed provider<br><br>Duration Limit per prescription:<br>• 60 days<br><br>Other requirements:<br>• Only manual manipulation is medically necessary<br>• Services must be expected to result in improvement in condition	Condition requirements:<br>• Chronic pain (duration >3 months)<br>• Chronic pain (duration >3 months) as part of comprehensive pain management program<br>• Nausea<br><br>Quantity Limits:<br>• 6 visits/year<br>• 16 visits/year combined chiropractic care and acupuncture<br>• 20 visits/year<br>• 204 units/year; 408 units/year combined acupuncture, chiropractic and/or massage therapy services<br><br>Provider requirements:<br>• Licensed network acupuncturists<br><br>Other requirements:<br>• Step requirements- failure of physical therapy, NSAIDs, muscle relaxants, analgesics

© 2018 Heyward J et al. *JAMA Network Open.*

• Expected improvement within 1 month of initiation		

© 2018 Heyward J et al. *JAMA Network Open.*

eREFERENCES

1. Rudd RA, Seth P, David F, Scholl L. Increases in Drug and Opioid-Involved Overdose Deaths—United States, 2010-2015. MMWR Morb Mortal Wkly Rep. 2016;65:1445–1452.

2. Kaiser Family Foundation. Medicaid MCO Enrollment. March 2017. Accessible at: https://www.kff.org/other/state-indicator/medicaid-enrollment-by-mco/. (Accessed December 21, 2017).

3. Kaiser Family Foundation. Medicare Advantage 2017 Spotlight: Enrollment Market Update. June 2017. Accessible at: https://www.kff.org/medicare/issue-brief/medicare-advantage-2017-spotlight-enrollment-market-update/. (Accessed December 21, 2017).

4. Houchens PR, Clarkson J, Herbold JS, Fohl H. "2015 Commercial Health Insurance: Overview of Financial Results." Milliman. March 2017. Accessible at: www.milliman.com/insight/2017/2015-commercial-health-insurance-Overview-of-financial-results/. (Accessed on December 20, 2017).

5. Kaiser Family Foundation. Health Insurance Market Competitiveness. 2014. Accessible at: https://www.kff.org/state-category/health-insurance-managed-care/insurance-market-competitiveness/. (Accessed December 21, 2017).

© 2018 Heyward J et al. *JAMA Network Open.*
